# *MolProbity*: all-atom structure validation for macromolecular crystallography

**DOI:** 10.1107/S0907444909042073

**Published:** 2009-12-21

**Authors:** Vincent B. Chen, W. Bryan Arendall, Jeffrey J. Headd, Daniel A. Keedy, Robert M. Immormino, Gary J. Kapral, Laura W. Murray, Jane S. Richardson, David C. Richardson

**Affiliations:** a Department of Biochemistry, Duke University, Durham, NC 27710, USA

**Keywords:** all-atom contacts, clashscore, automated correction, *KiNG*, ribose pucker, Ramachandran plots, side-chain rotamers, model quality, systematic errors, database improvement

## Abstract

*MolProbity* is a structure-validation web service that provides broad-spectrum solidly based evaluation of model quality at both the global and local levels for both proteins and nucleic acids. It relies heavily on the power and sensitivity provided by optimized hydrogen placement and all-atom contact analysis, complemented by updated versions of covalent-geometry and torsion-angle criteria. Some of the local corrections can be performed automatically in *MolProbity* and all of the diagnostics are presented in chart and graphical forms that help guide manual rebuilding. X-ray crystallography provides a wealth of biologically important molecular data in the form of atomic three-dimensional structures of proteins, nucleic acids and increasingly large complexes in multiple forms and states. Advances in automation, in everything from crystallization to data collection to phasing to model building to refinement, have made solving a structure using crystallo­graphy easier than ever. However, despite these improvements, local errors that can affect biological interpretation are widespread at low resolution and even high-resolution structures nearly all contain at least a few local errors such as Ramachandran outliers, flipped branched protein side chains and incorrect sugar puckers. It is critical both for the crystallographer and for the end user that there are easy and reliable methods to diagnose and correct these sorts of errors in structures. *MolProbity* is the authors’ contribution to helping solve this problem and this article reviews its general capabilities, reports on recent enhancements and usage, and presents evidence that the resulting improvements are now beneficially affecting the global database.

## Abstract 2

*MolProbity* structure validation will diagnose most local errors in macromolecular crystal structures and help to guide their correction.

## 1. Summary of *MolProbity* flow and user interactions

The usual interaction with *MolProbity* (Davis *et al.*, 2007 ▶) is through the internet at http://molprobity.biochem.duke.edu or as a main menu item on our general laboratory website at http://kinemage.biochem.duke.edu. [For bulk users, it is also possible to set up your own local *MolProbity* server or to use the individual programs in command-line mode.] Tutorial exercises for the whole process of diagnosing and fixing errors can be found on the kinemage site under Teaching/MolProbity.

A typical *MolProbity* session starts with the user uploading a coordinate file of their own or fetching one from the PDB or NDB databases (Berman *et al.*, 1992 ▶, 2000 ▶) in new or old PDB format or in mmCIF format. After checking the thumbnail image and listed characteristics of the input file and editing or reloading if needed, H atoms are added and optimized, with automated correction of Asn/Gln/His 180° flips if needed (Word, Lovell, Richardson *et al.*, 1999 ▶).

**Figure 1:**
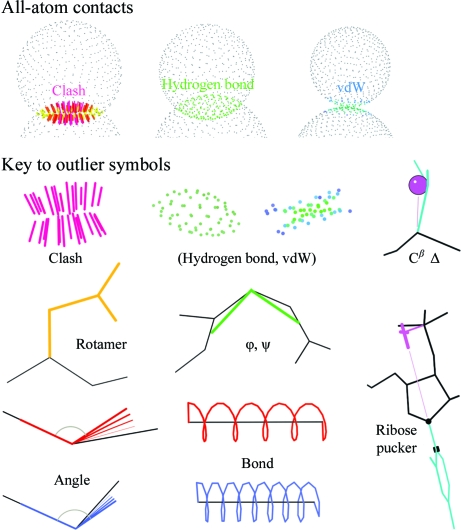
An outlier legend, showing each symbol used in a *MolProbity* multi-criterion kinemage and illustrating the relationship of the three types of all-atom contact to the atomic van der Waals (vdW) surfaces (spheres of small gray dots). The symbols for favorable hydrogen bonds and vdW contacts are included for completeness, as well as the hot-pink spikes of a clash outlier. A C^β^ deviation of ≥0.25 Å is shown as a magenta ball centered on the ideal C^β^ position and tangent to the modeled position. Bad rotamers are shown as gold side chains and Ramachandran outliers as heavy green lines to the midpoints of the two peptides. Bond-angle outliers are indicated by a fan of lines from the ideal to the modeled bond (red if wide, blue if narrow). Bond-length outliers are indicated as stretched (red) or compressed (blue) springs. A suspicious ribose pucker is diagnosed by the perpendicular distance from the 3′ (following) phosphate to the line of the glycosidic C1′—N1/9 bond and is flagged by a representation of that construction (in magenta if too short, as here, and in purple if too long).

The user then chooses which validation analyses to run and what reports and output files to generate. The *MolProbity* interface adjusts the defaults and options presented and even the page flow depending on user choices and on the properties of the file being worked on. These adjustments make *MolProbity* simple for novice users, while at the same time allowing advanced users to have great control over their runs. The core ‘glue’ that generates the HMTL code controlling the main user interface and programmatic interactions of *MolProbity* is implemented in the PHP programming language. Underlying the PHP core, the majority of the analysis tasks in *MolProbity* are performed by individual programs written in a range of languages, including C, C++, Java and Perl. It uses *REDUCE* and *PROBE* for all-atom contact analysis, *RAMALYZE*, *ROTALYZE*, *DANGLE*, *SILK* and *SUITENAME* for other criteria and *KiNG* for three-dimensional visualization of the structure and its validation markers directly in the browser. Fig. 1 ▶ shows a key to *MolProbity*’s graphical markers for validation outliers. Further details are provided below on the specific analyses that *MolProbity* can perform. The validation results are reported in the form of summaries, charts, two-dimensional and three-dimensional graphics and output files for download.

**Figure 2:**
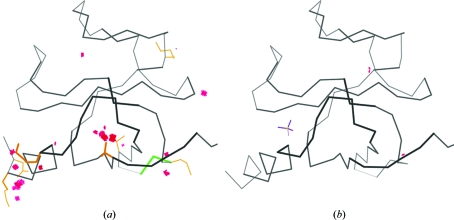
Two multi-criterion validation kinemages illustrating the successful outcome of an overall process of *MolProbity* diagnosis and structure improvement. (*a*) The original 1lpl Cap-Gly structure (Li *et al.*, 2002 ▶) shows three major clusters of clash, rotamer and Ramachandran problems plus a few isolated outliers. (*b*) The corrected 1tov structure (Arendall *et al.*, 2005 ▶) has essentially no outliers, a 4% lower *R*_free_, a bound sulfate and an additional turn of helix at the N-terminus.

The crucial final step in the *MolProbity* process is for the crystallographer to download the result files and work off-line to correct as many of the diagnosed problems as feasible. Rebuilding with consideration of the validation outliers, the electron density and the surrounding model is usually per­formed either in *Coot* (Emsley & Cowtan, 2004 ▶) or in *KiNG* (Chen *et al.*, 2009 ▶). At resolutions of about 2.5 Å or better it is possible to correct the great majority of outliers (Arendall *et al.*, 2005 ▶), with an order-of-magnitude improvement in the various *MolProbity* scores and some improvement in geometry, map quality, *R* factor and *R*_free_. An example is shown in Fig. 2 ▶ with before-and-after multi-criterion kinemages.

## 2. Validation analyses

### 2.1. Addition of H atoms

The presence of H atoms (both nonpolar and polar) is a critical pre­requisite for all-atom contact analysis. Although refinement using H atoms is becoming more common, most crystal structures are still deposited without H atoms. Once a PDB structure file has been uploaded, *MolProbity* detects whether the file contains a suitable number of H atoms; if not, then the ‘Add H atoms’ option is presented to users first. *MolProbity* uses the software *REDUCE* (Word, Lovell, Richardson *et al.*, 1999 ▶) to add and optimize hydrogen positions in both protein and nucleic acid structures, including ligands, but does not add explicit H atoms to waters. OH, SH and NH_3_ groups (but not methyl groups) are rotationally optimized and His protonation is chosen within each local hydrogen-bond network, including interactions with the first shell of explicit waters.

**Figure 3:**
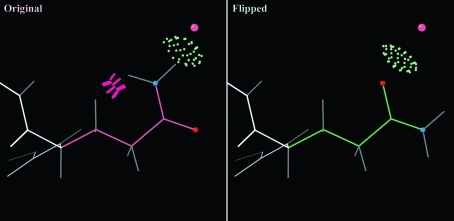
The simple ‘flip’ correction of a Gln side-chain amide in the 2dq4 threonine 3-dehydrogenase structure (R. Omi, T. Yao, M. Goto, I. Miyahara & K. Hirotsu, unpublished work), a better-than-average 2.5 Å resolution structure. Both orientations make a hydrogen bond to the crystallographic water, but the original has a serious internal clash of the NH_2_ group with its own C^β^ hydrogen.

A common problem is that the side-chain ends of Asn, Gln and His are easily fitted 180° backwards, since the electron density alone cannot usually distinguish the correct choice of orientation. *REDUCE* can automatically diagnose and correct these types of systematic errors by considering all-atom steric overlaps as well as hydrogen bonding within each local network. Automatic correction of Asn/Gln/His flips is the default option in *MolProbity* during addition of H atoms. *MolProbity* presents each potential flip correction to the user in kinemage view so they have the option of inspecting the before-and-after effects of each flip and approving (or rejecting) each correction. Fig. 3 ▶ shows an example of a simple Gln flip that is unquestionably correct but that could not have been decided on the basis of hydrogen bonding alone. Other examples can be much more complex, with rotatable OH positions, large hydrogen-bond net­works and multiple com­peting inter­actions evaluated exhaustively.

Users can also choose to add H atoms without Asn/Gln/His flips, which is useful in evaluating the atomic coordinates as they were deposited, but which rejects the easiest and most robustly correct improvement that can be made in a crystallo­graphic model (Word, Lovell, Richardson *et al.*, 1999 ▶; Higman *et al.*, 2004 ▶). If flips are performed, the user needs to download and use the corrected PDB file (either with or without the H atoms) in order to benefit.

### 2.2. All-atom contact analysis

Once H atoms have been added to (or detected in) a structure, then the complete ‘Analyze all-atom contacts and geometry’ option is enabled. A main feature of this option is the all-atom contact analysis, which is performed by the program *PROBE* (Word, Lovell, LaBean *et al.*, 1999 ▶). *PROBE* operates by, in effect, rolling a 0.5 Å diameter ball around the van der Waals surfaces of atoms to measure the amount of overlap between pairs of nonbonded atoms. When non-donor–acceptor atoms overlap by more than 0.4 Å, *PROBE* denotes the contact as a serious clash, which is included in the reported clashscore and is shown in kinemage format as a cluster of hot-pink spikes in the overlap region (Fig. 1 ▶). Such large overlaps cannot occur in the actual molecule, but mean that at least one of the two atoms is modeled incorrectly. *MolProbity* allows users to select any combination of clashes, hydrogen bonds and van der Waals contacts to calculate and display on the structure. By default, all three are enabled for structures that are not excessively large; for large structures, van der Waals contacts are deselected.

**Figure 4:**
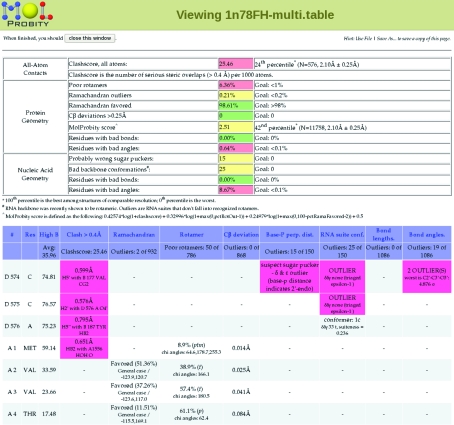
A *MolProbity* results summary and sortable multi-criterion chart for the 1n78 Glu tRNA-synthetase complex at 2.1 Å resolution (Sekine *et al.*, 2003 ▶). The summary gives numerical values, goals and relative percentiles for clashscore, torsion angle and geometry criteria for both protein and nucleic acid components, with traffic light color-coding for good and bad values. Below the summary is a short extract from the detailed chart with values and specifics for each criterion on each residue. Notice that outliers (highlighted) tend to cluster.

The ‘clashscore’ is the number of serious clashes per 1000 atoms. It is reported in the *MolProbity* summary (top of Fig. 4 ▶), with a red/yellow/green color coding for absolute quality. The structure’s percentile rank for clashscore value within the relevant resolution range is also given. In the detailed sortable ‘multi-chart’ (an extract is shown below the summary in Fig. 4 ▶), the worst clash ≥0.4 Å is listed for each residue and highlighted in pink.

### 2.3. Torsion-angle combinations: updated Ramachandran and rotamer analyses

Also included in the ‘Analyze all-atom contacts and geometry’ option is an evaluation of where residues fall in the multi-dimensional distributions of Ramachandran backbone ϕ, ψ angles and side-chain rotamer χ angles. The reference distributions are currently from 100 000 residues in 500 files, quality-filtered at both the file and the residue level. The Ramachandran plots are separated for Gly, Pro and pre-Pro residue types; the general plot has only one in 2000 residues outside the ‘allowed’ contour, which is the same probability as a 3.5σ outlier in a normal distribution. The three specific plots can be robustly contoured only down to excluding one in 500 residues (about 3σ) in the current reference data, but will soon be updated. By ‘robust’ we mean that the contour does not shift with further improvement in resolution or *B* or with different subselections of the data. When values plateau in this way we can define clear absolute goals for the measure, such as 98% for Ramachandran favored, <0.2% for Ramachandran outliers, <1% for poor rotamers and 0 for C^β^ deviation outliers; other goal levels given in the summary are more arbitrary.

**Figure 5:**
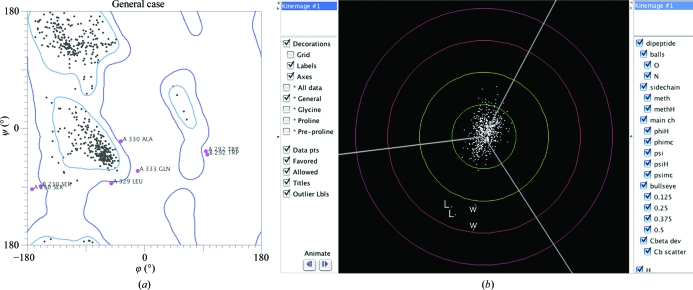
The general case Ramachandran kinemage and the C^β^ deviation kinemage for file 2dq4. In (*a*) the ϕ, ψ values for each residue are plotted on a background of the smoothed contours from high-quality data (see text). Over 98% lie inside the inner ‘favored’ 98% contour, but there are seven outliers outside the outer 99.95% contour. Gly, Pro and pre-Pro residues are on separate plots (not shown). In (*b*) the C^β^ deviation kinemage shows each residue’s C^β^ position relative to an ideal C^β^ and its three bond vectors (gray lines). Circles mark the deviation distances, with the yellow circle at the 0.25 Å cutoff for outliers. Most of the distribution is good, but an adjacent Leu and Trp in each chain (labeled) are part of an outlier cluster and probably reflect distortions caused by a local fitting problem.

However, Ramachandran outliers are of course not rare in general; they increase as a function of resolution (Arendall *et al.*, 2005 ▶) and especially of *B* factor (Lovell *et al.*, 2003 ▶). Fig. 5 ▶(*a*) shows the points for an above-average 2.5 Å resolution structure plotted on the smoothed contours of the reference distribution for favored (enclosing 98% of the good data) and allowed (enclosing 99.95% of the good data). The four Ramachandran plots are presented in kinemage and pdf form, scores are given in the multi-criterion chart (Fig. 4 ▶) and outliers are flagged in green on the multi-criterion kinemage (Figs. 1 ▶ and 2 ▶).

Multi-dimensional rotamer distributions for the individual side-chain types are currently contoured only down to excluding 1% of the high-quality data. [All such distributions used in *MolProbity* will be periodically updated to take advantage of the expanding reference data.] These are updates of the ‘penultimate rotamer library’ (Lovell *et al.*, 2000 ▶), although certainly not yet ultimate. Above the 1% level sets of χ values are assigned to named rotamers; below 1% they are designated as poor rotamers, not outliers, since they are disfavored but quite possible if stabilized by tight packing or a couple of good hydrogen bonds. However, there is no justification for fitting poor rotamers on the protein surface with no interactions to hold them in an unfavorable conformation. Some bad rotamers result from systematic errors arising from fitting branched side chains (Thr/Val/Ile/Leu/Arg) backwards into ambiguous density. The two major systematic errors for Leu are described in Lovell *et al.* (2000 ▶) and are given zero rotamer-quality scores in *MolProbity*; other cases are discussed in §[Sec sec3.2]3.2. Poor rotamers are scored in the multi-criterion chart and are represented by gold side chains in the multi-criterion kinemages (Figs. 1 ▶ and 2 ▶). The *Mol­Probity* summary (top of Fig. 4 ▶) reports the percentage of residues with poor rotamers, Ramachandran outliers and Ramachandran favored conformations.

### 2.4. Covalent-geometry analyses

*MolProbity* now evaluates backbone bond-length and bond-angle outliers. Mean overall deviations in geometrical parameters are a measure of correct procedures and weights in refinement, but not of structural accuracy. Local geometry outliers ≥4σ, however, are most often the indirect result of local misfitting and are thus very useful diagnostics, especially for bond angles. [Note that some such problems occur even at very high resolution, either at the ends of alternate conformations or in under-restrained regions with high *B* factor.] A major recent addition to *MolProbity* is the ability to assess the ideality of covalent backbone geometry for protein and RNA with the Java program *DANGLE*, which is also available for standalone command-line use from the software section at http://kinemage.biochem.duke.edu. New and more intuitive visualizations for backbone bond-length and angle deviations have been developed for display on the three-dimensional structure. For bond lengths, a ‘spring’ is drawn along the bond axis and scaled such that the ideal distance is equivalent to six turns and 4σ deviation corresponds to one turn of stretching or compaction (Fig. 1 ▶). For bond angles, bold lines are drawn to represent the ideal angle and a fan of increasingly thin lines fades out across the model *versus* ideal angle difference to highlight the extent of the deviation (Fig. 1 ▶). In each case model values greater (or less) than the corresponding ideal value by at least 4σ are displayed in red (or blue) and are listed on the multi-criterion chart. Protein backbone parameters were derived from Engh & Huber (2001 ▶) and nucleic acid backbone parameters from Parkinson *et al.* (1996 ▶).

The C^α^ atom is where local problems with backbone or side-chain fitting must be reconciled. This affects the bond angles or improper dihedrals that define the C^β^ position, but can be manifested in almost any combination of those individual parameters. Therefore, *MolProbity* evaluates the resulting overall distortion of the C^β^ position from ideality, called the C^β^ deviation (Lovell *et al.*, 2003 ▶). Residues with a C^β^ deviation of ≥0.25 Å are flagged in the chart and shown in the kinemage as a magenta ball centered on the ideal position (calculated from the backbone coordinates and allowing for changes in the τ angle) and tangent to the modeled position (Fig. 1 ▶), since we have found that values of ≥0.25 Å are very often correlated with some form of local misfitting. Fig. 5 ▶(*b*) shows a separate plot produced for all the C^β^ deviation values in a structure, shown relative to the ideal C^β^ position. The Leu and Trp C^β^ outliers in each chain form a tight turn with a suspicious peptide orientation and eight other outliers and so must represent some form of misfitting.

### 2.5. Nucleic acid analyses

Nucleic acids are treatable equivalently to proteins for all-atom contacts and clashscore and for bond-length and bond-angle analyses, as long as the correct parameter sets are used. Both DNA and RNA show more non-uniform distribution of local problems than do proteins, with the bases and phosphates located well and the rest of the sugar-phosphate backbone very prone to errors (Word, Lovell, Richardson *et al.*, 1999 ▶; Murray *et al.*, 2003 ▶), since it has many torsion variables and rather indistinct electron density at moderate resolutions. All-atom contacts are very helpful in diagnosing backbone misfitting, especially for RNA structures, which are rapidly gaining biological interest and structural attention.

**Figure 6:**
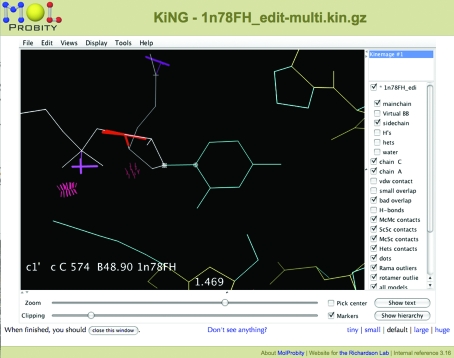
Close-up of a ribose-pucker outlier in the multi-criterion kinemage for 1n78, with backbone and bases turned on. C574 has a short phosphate-to-glycosidic bond perpendicular (magenta line and cross), but was fitted with an intermediate pucker near C3′-*endo*. The bad pucker torques the connected groups strongly, probably causing the bond-angle outliers (red) and steric clashes (hot-pink spikes). Note that C574 is in the binding interface between RNA (white backbone) and enzyme (yellow backbone) close to the active site.

In addition, *MolProbity* now includes diagnosis of suspect ribose puckers and torsion-angle analysis of preferred RNA backbone conformers. We have found that the dominant C3′-­*endo* and C2′-*endo* sugar puckers are highly correlated to the perpendicular distance between the C1′–N1/9 glycosidic bond vector and the following (3′) phosphate: >2.9 Å for C3′-­*endo* and <2.9 Å for C2′-*endo*. *MolProbity* checks this distance against the modeled sugar pucker, as well as outliers in individual ∊ or δ values. All such outliers are listed in the multi-chart and ribose-pucker outliers are flagged in the kinemage (Fig. 1 ▶). An example is shown in Fig. 6 ▶, where what should have been a C2′-*endo* pucker (by the short perpendicular) was fitted as an intermediate unfavorable pucker close to the more common default C3′-*endo* pucker, also producing geometry and ∊ outliers.

High-dimensional analysis of the combinations of backbone torsion angles within an RNA ‘suite’ (the unit from sugar to sugar) has shown that there are distinct ‘rotameric’ backbone conformers. The RNA Ontology Consortium has defined a two-character nomenclature and an initial set of 54 favorable RNA backbone conformers (Richardson *et al.*, 2008 ▶). We created the *SUITENAME* program to identify either the named conformer or an outlier for each suite in an RNA structure. These conformers and their ‘suiteness’ quality score are listed in the *MolProbity* multi-chart.

### 2.6. The overall *MolProbity* score

In response to user demand, the ‘*MolProbity* score’ provides a single number that represents the central *Mol­Probity* protein quality statistics. It is a log-weighted combination of the clashscore, percentage Ramachandran not favored and percentage bad side-chain rotamers, giving one number that reflects the crystallographic resolution at which those values would be expected. Therefore, a structure with a numerically lower *MolProbity* score than its actual crystallo­graphic resolution is, quality-wise, better than the average structure at that resolution. There is some distortion in the fit at very high or very low resolutions; for these ranges it is preferable to judge by the resolution-specific percentile score, which is also reported in the summary. Percentile scores are currently given for clashscore and for *MolProbity* score relative to the cohort of PDB structures within 0.25 Å of the file’s resolution.

## 3. Correction of outliers

### 3.1. Manual rebuilding

**Figure 7:**
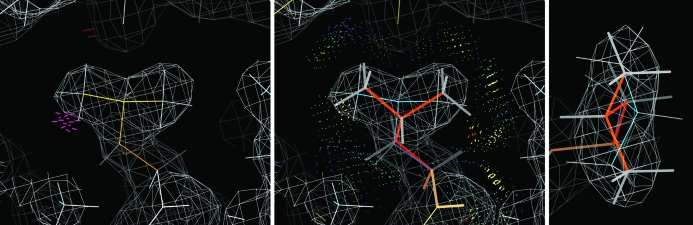
Rebuilding of a backward-fitted Leu side chain in *KiNG* off-line in the DNA polymerase 1xwl at 1.7 Å resolution (Kiefer *et al.*, 1997 ▶). The original (left) fits the density fairly well but is a rotamer outlier with a clash. One of the two best Leu rotamers also fits the density well with good all-atom packing. The top view (right) shows the 180° relationship of the two conformations.

Except for Asn/Gln/His flip corrections, *MolProbity* does not yet directly include the ability to correct the errors it finds in structures; it relies on users having access to standalone local software for rebuilding and refinement. The stand­alone version of *KiNG* has some rebuilding tools for modeling side chains and making small local ‘backrub’ adjustments to structures, with the help of electron-density display, interactive contact dots and rotamer evaluation (Davis *et al.*, 2006 ▶; Chen *et al.*, 2009 ▶). Fig. 7 ▶ illustrates such a correction process in *KiNG*, rebuilding a backward-fitted leucine with a clash and a bad rotamer (one of the cases of a systematic error), resulting in an ideal geometry side chain with an excellent rotamer and well packed all-atom contacts. The top view shows that the original and rebuilt side chains fit the terminal methyls into the same rather ambiguous density, but move the C^γ^ substantially. More recent versions of this DNA polymerase structure (*e.g.* PDB code 2hhv at 1.55 Å resolution; Warren *et al.*, 2006 ▶) all use the new conformation. Manual rebuilding is facilitated by the fact that all-atom clashes are inherently directional, as are bond-angle distortions, while a good library of rotamer choices helps the user test all the alternatives.

For more extensive refitting, a fully featured crystallo­graphic rebuilding program such as *Coot* (Emsley & Cowtan, 2004 ▶) is needed. *MolProbity* generates ‘to-do’ scripts that can be read into *Coot*, bringing up a button list, where each entry will zoom to a problem area. In combination with the ability of *Coot* to use *REDUCE* and *PROBE* interactively to generate all-atom contact dots, these features make it easier to address the problems diagnosed by *MolProbity*. Any rebuilding that moves atoms must of course then undergo further crystallo­graphic refinement. Our own laboratory tested the combined cycle of *MolProbity*, rebuilding and refinement on about 30 protein structures as part of the SouthEast Collaboratory for Structural Genomics (Arendall *et al.*, 2005 ▶), finding that its early application led to a smoother structure-solution process and demonstrably better final structures. In addition to backward-fitted side chains, commonly corrected problems included peptide flips, switched backbone and side chain near chain ends, ‘waters’ that were really ions, noise peaks or unfit alternate conformations and occasionally a shift in sequence register. Many other crystallographic groups have since adopted these methods.

### 3.2. Automated corrections

For correcting RNA-suite outliers, we have collaboratively developed the independent program *RNABC* (Wang *et al.*, 2008 ▶), which performs an automated search for more suitable backbone conformations of an RNA suite diagnosed with a bad ribose pucker or serious clashes. It leaves the more accurately determined bases and P atoms fixed in place and performs a pruned but systematic search through the other parameters, outputting all acceptable alternatives found within user-set tolerance limits.

Recently, we have developed and tested the *AUTOFIX* program for automated correction of diagnosed backward-fitted Thr, Val, Leu and Arg side chains (Headd *et al.*, 2009 ▶). In contrast to Asn/Gln/His flips, which simply exchange atoms and do not change the agreement with the data, these more complex side chains require real-space refinement in order to determine the proper correction and crystallographic re-refinement after the approximate 180° flips have been made. The original version used *Coot* to perform rotamer selection and real-space refinement for the proposed corrections, with *MolProbity* diagnosis before and after. Results were checked by re-refinement. Run on a sample of 945 PDB files, *AUTOFIX* accepted corrections for over 40% of diagnosed bad Thr, Val and Leu side chains and 15% of bad Arg side chains, or 3679 corrected side chains. A second version is now in the testing stage that substitutes *PHENIX* real-space refinement, has a faster Python wrapper and also works on Ile. It will soon be incorporated into *MolProbity*. The most important of our requirements for *AUTOFIX* is that it does no harm; we are willing to miss some of the possible corrections in order to ensure that those we accept are essentially always true improvements. *AUTOFIX* should provide *MolProbity* users with an easy and reliable way of making an initial set of meaningful improvements to their protein structures. Thr and Arg, in particular, make hydrogen bonds that are often important at active sites or binding interfaces and since they are asymmetrical these interactions change drastically if the side chain is fitted backwards. Such improvements were often seen in the test set.

## 4. Other *MolProbity* utility functions

### 4.1. Interface analysis

*PROBE* can also be used to calculate the all-atom contacts at interfaces, *e.g.* between two chains of a structure or between a protein and a ligand. Access to this feature is provided in *MolProbity* by the ‘Visualize interface contacts’ analysis option after H atoms have been added. The user is required to choose the chains and/or the molecular types for which to calculate the contacts (*e.g.* protein *versus* protein or protein *versus* heteroatoms or RNA). This functionality creates both a kinemage with the resulting all-atom contacts displayed on the model and a text list of the atom pairs in contact.

### 4.2. Protein loop fitting

*MolProbity* includes the Java software *JIFFILOOP* for providing potential protein-fragment conformations that can fit within a gap in a protein structure. We have defined a seven-parameter system that describes the spatial relationship between any two peptides. Briefly, this system consists of the sequence separation, the distance between the two inner C^α^ atoms, two pseudo-angles and three pseudo-dihedrals. We used this system to create a library of *B*-factor-filtered fragments from one to 15 peptides long from our Top5200 database of structures, a set of structures chosen from each 70% nonredundant group defined by the PDB, requiring an average of resolution and *MolProbity* score of ≤2.0. *MolProbity* runs *JIFFILOOP* to search this library for candidate fragments to fill gaps within a structure. Alternatively, users can enter beginning and ending residue numbers and *MolProbity* will search for fragments which can fit between those two residues. Because this process can be fairly time-intensive, *JIFFILOOP* is not listed under ‘Suggested Tools’ and is currently only accessible under ‘All Tools’ or at the Site map. Also, owing to the size of this package it must be added separately to the installation for a standalone *MolProbity* server.

### 4.3. Kinemage construction and viewing

*MolProbity* provides scripts (under the ‘Make simple kinemages’ option) for constructing a number of commonly used kinemage three-dimensional interactive visualization options such as ribbons and various types of stick figures. This functionality is useful for quick browsing of a structure or for initial creation of an illustration or presentation. The file-input page can also accept upload of pre-existing kinemage files for direct on-line viewing within the built-in kinemage viewer *KiNG*.

### 4.4. Other file types and functions

*MolProbity* uses a built-in PDB ‘het_dictionary’ for the information needed to add H atoms to small-molecule ligands. The user can construct and read in a custom dictionary if their file contains novel ligands. There is also provision for either uploading or fetching an electron-density map from the Electron Density Server (Kleywegt *et al.*, 2004 ▶) in any of several formats to view on-line in *KiNG* together with the model and validation results. To investigate functional sites that span across asymmetric units, one can fetch a biological unit file from the PDB. In the file-editing feature, the user can specify whether multiple ‘models’ are alternatives (as in an NMR ensemble) or have been pressed into service for the extra chains in the biological unit. Some X-ray structures are now treated as ensembles. For such cases, *MolProbity* internally splits the models and analyzes them separately, but constructs an outlier summary strip-chart and a multi-model multi-criterion validation kinemage with both the models and their features under on/off button control. File editing also allows the deletion of chains either before or after hydrogen placement, specifying the resolution of the structure if not given in the file header or removing unwanted H atoms. These tools make it easier and faster to analyze particular parts of a structure using *MolProbity* and they help to maintain compatibility with other older software. These options are always available as separate utility functions, independent of validation or hydrogen content.

### 4.5. PDB-format interconversion

The release of the remediated PDB version 3.0 format in August 2007 included a number of significant changes, particularly to H-atom names and to nucleic acid residue and atom names. In order to maintain compatibility with the PDB, we converted the entire *MolProbity* core to use the new format by default. This included updating *REDUCE*, *PROBE*, *KiNG* and *PREKIN*. However, we realised that users might need to analyze files that were still in the older PDB version 2.3 format. In order to maintain backwards compatibility, we created a Remediator script (available as a standalone Perl or Python script) that can interconvert between the old and the new PDB formats. Whenever a file is input, *MolProbity* will scan for the presence of old-format atom names and if it detects any then it will run the Remediator script to automatically convert the input file to the new format. After analysis there is then an option available to run the Remediator script and downgrade the output file back into the old version 2.3 format if needed. This allows use of the *MolProbity* analysis tools even together with older software that has not been updated to use the new format.

## 5. Discussion

### 5.1. Global *versus* local, absolute *versus* comparative

There are three quite different purposes served by structure validation: a gatekeeper function on quality for reviewers or organizations, an aid to crystallographers for obtaining the most model accuracy from their data and a guide to end users for choosing appropriate structures and confidence levels for the conclusions they want to draw.

Validation criteria also come in distinct flavors. Those based on the diffraction data are inherently global with respect to the model; for instance, resolution (which is still the most valuable single-factor estimate of model accuracy) and *R*_free_ (Brünger, 1992 ▶). On the data side, there are also gatekeeper checks for unusual problems such as twinning or gross data incompleteness. R.m.s.d. or r.m.s.*Z*. of deviations from geo­metrical target values are global, but they only evaluate procedural aspects of refinement and have little to do with model accuracy. Most other validation criteria are inherently local (at the residue or even atom level), including *B* factor, real-space measures such as RSR-*Z* (Kleywegt *et al.*, 2004 ▶) and model-only measures such as the various *MolProbity* criteria described here. Any local measure becomes global when expressed in some normalized form across the entire structure, such as an average, a distribution match or a percentage occurrence of outliers.

Strictly local measures are usually not resolution-dependent, but their globally defined versions often are. For some purposes, the desirable form of measure is a comparison (usually a percentile rank) with the cohort of PDB structures at similar resolution. *MolProbity* currently provides resolution-group percentiles for clashscore and for *MolProbity* score and will probably expand that to other criteria.

Reviewer/gatekeepers are primarily interested in global relative measures such as resolution-dependent percentiles and to some extent in absolute local flags for judging the support behind specific claims. Crystallographers need global relative measures to judge how well they have made use of their data, but it is the local measures, especially specific outliers, that are crucial to helping them to achieve a more accurate structure and to avoid making any dubious claims in poor local regions (such as an invisible inhibitor). End users need absolute global measures to choose between structures and absolute local measures to judge the reliability of the particular features they find of interest.

Because of the importance of improving and evaluating the accuracy of individual details of biological importance, both in each structure and in the database as a whole, we have chosen in *MolProbity* to emphasize calculation and user-friendly display of local indicators. We have also tried to minimize ‘false alarms’, so that a flagged outlier is almost always worth a close look.

### 5.2. Impact on database quality

Since *MolProbity* was first made available in late 2002, serious user work sessions (performing some operation on an input coordinate file) have multiplied by a large factor each year, with a cumulative total that is now approaching 100 000 by thousands of distinct users. In addition, many companies and structural genomics centers run their own *MolProbity* servers internally and some aspects have been incorporated into other software or meta-servers. 80% of *MolProbity* input files are uploaded, presumably by working structural biologists, and the rest are fetched from databases, presumably by end users. Those end users also include students, since *MolProbity* is increasingly being used for instructional exercises in bio­chemistry classes from high school to graduate level.

*MolProbity*’s unique feature is clash analysis from all-atom contacts, which provides sensitive new evaluation independent of refinement targets. Not surprisingly, the average clashscore remained constant (either globally or by resolution) up to 2002, since there was then no feasible way of targeting or even measuring all-atom clashes. The percentage of incorrect Asn/Gln/His flips also remained level or rose slightly prior to 2003, despite the availability of a hydrogen-bond-based system in *WHAT IF* (Hooft *et al.*, 1996 ▶), and even while refinement methods, automation and Ramachandran and rotamer quality all improved.

**Figure 8:**
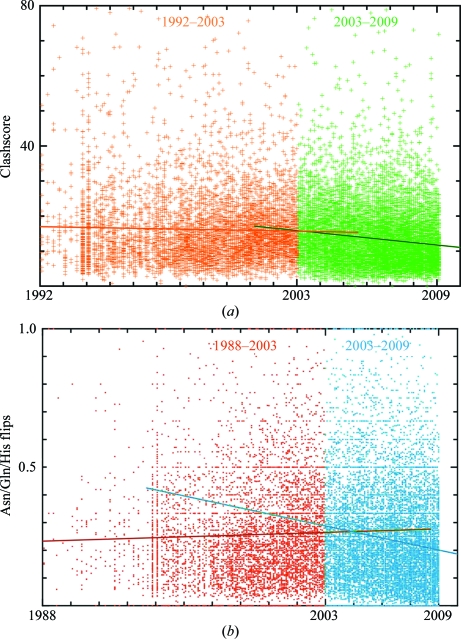
*MolProbity*-relevant quality criteria as a function of time for all structures in the PDB at a middle range of resolution, separately fitted before and after introduction of the web site. (*a*) All-atom clashscore (see §[Sec sec2.2]2.2); (*b*) percentage of Asn/Gln/His flips (see §[Sec sec2.1]2.1).

To evaluate the contribution *MolProbity* has made to crystallographic model quality in general, we have therefore plotted clashscore and Asn/Gln/His flips as a function of time in Fig. 8 ▶, with separate linear fits before and after the end of 2002. Gratifyingly, in both cases there is a clear trend of improvement since 2003. Median values also improve very steadily over that period. Anecdotal evidence indicates that this trend is mainly a consequence of thorough adoption of *MolProbity*-based methods by a small but growing fraction of crystallographers and there is therefore still much scope for further improvement in the future.

## 6. *MolProbity* availability

*MolProbity* is freely available for download from http://molprobity.biochem.duke.edu for use as a local server. This option requires either Linux or MacOSX, together with PHP and Apache. Instructions for installing *MolProbity* locally are included with the download. Having a local install allows users to access the *MolProbity* analysis tools without internet access, as well as allowing companies with privacy or confidentiality concerns to use *MolProbity*. However, one of the most significant advantages to having a local installation of *Mol­Probity* is access to command-line tools. These tools provide access to the major analysis tools in *MolProbity* without having to use the web interface. Also, several scripts are included which allow users to run *MolProbity* analysis on a set of files rather than just one at a time. Some of the more useful command-line scripts include the following: scripts for adding H atoms, with or without flips, a script for obtaining overall scores for a set of files and a script for calculating a residue-by-residue analysis of a structure.

For users of the *PHENIX* crystallography system (Adams *et al.*, 2002 ▶, 2009 ▶), a number of the main *MolProbity* quality-analysis tools have been incorporated directly into *PHENIX* and are accessible through command-line tools or in the *PHENIX* GUI, including *REDUCE*, *PROBE*, *RAMALYZE*, *ROTALYZE*, *CBETADEV* and *CLASHSCORE*. Currently, only tabular results are provided; we are exploring the possibility of incorporating *KiNG* and validation visualizations into *PHENIX*.

All of the individual programs called by *MolProbity* are also available, multi-platform and open source, from the software section at http://kinemage.biochem.duke.edu.
